# Theranostic applications of optical coherence tomography in neurosurgery?

**DOI:** 10.1007/s10143-021-01599-x

**Published:** 2021-08-16

**Authors:** Karl Hartmann, Klaus-Peter Stein, Belal Neyazi, I. Erol Sandalcioglu

**Affiliations:** Universitätsklinik Für Neurochirurgie, Otto-Von-Guericke-Universität Magdeburg, Leipziger Str. 44, 39120 Magdeburg, Deutschland

**Keywords:** Optical coherence tomography, Microscope integration, Neurosurgery, Intraoperative imaging

## Abstract

In light of our own experiences, we value the existing literature to critically point out possible “near” future applications of optical coherence tomography (OCT) as an intraoperative neurosurgical guidance tool. “Pub Med”, “Cochrane Library”, “Crossref Metadata Search”, and “IEEE Xplore” databases as well as the search engine “Google Scholar” were screened for “optical coherence tomography + neurosurgery”, “optical coherence tomography + intraoperative imaging + neurosurgery”, and “microscope integrated optical coherence tomography + neurosurgery”. n = 51 articles related to the use of OCT as an imaging technique in the field of neurosurgery or neurosurgical research. n = 7 articles documented the intraoperative use of OCT in patients. n = 4 articles documented the use of microscope-integrated optical coherence tomography as a neurosurgical guidance tool. **The Results demonstrate that** OCT is the first imaging technique to study microanatomy in vivo. Postoperative analysis of intraoperative scans holds promise to enrich our physiological and pathophysiological understanding of the human brain. No data exists to prove that OCT-guided surgery minimizes perioperative morbidity or extends tumor resection. But results suggest that regular use of microscope-integrated OCT could **increase security** during certain critical microsurgical steps like, e.g., dural dissection at cavernous sinus, transtentorial approaches, or aneurysm clip placement. **Endoscopy integration** could aid surgery in regions which are not yet accessible to real-time imaging modalities like the ventricles or hypophysis. **Theranostic instruments** which combine OCT with laser ablation might gain importance in the emerging field of minimal invasive tumor surgery. OCT depicts vessel wall layers and its pathologies uniquely. Doppler OCT could further visualize blood flow in parallel. These abilities shed light on promising future applications in the field of **vascular neurosurgery**.

## Introduction

Microneurosurgery remains an exceedingly demanding and dexterous fine motor task. Microscope-integrated three-dimensional imaging techniques which delineate the microstructural composition of tissue in the field of view are missing so far.

OCT imaging depends on the detection of back-scattered near-infrared light and is therefore harmless to biological tissue [3]. Notably OCT offers an outstanding axial spatial resolution from 1 to 15 μm. Among in vivo imaging methods, it remains unprecedented and approaches spatial resolution of conventional histopathology [10]. Penetrating depths depend on the optical tissue density. They range from 4 mm in air to 2.5 mm in dens tissue. With approximately 3.1 mm in the human cerebral cortex OCT well meets microsurgical requirements [9].

Physically depending on light microscope integration is fairly simple [25]. This opens up the ability of contact free three-dimensional, real-time scanning of tissue in the field of view during microsurgical procedures [26]. In ophthalmology, the technique yet proved robustness and is daily integrated in vitreoretinal surgical setups [19, 36].

**Fig. 1:**
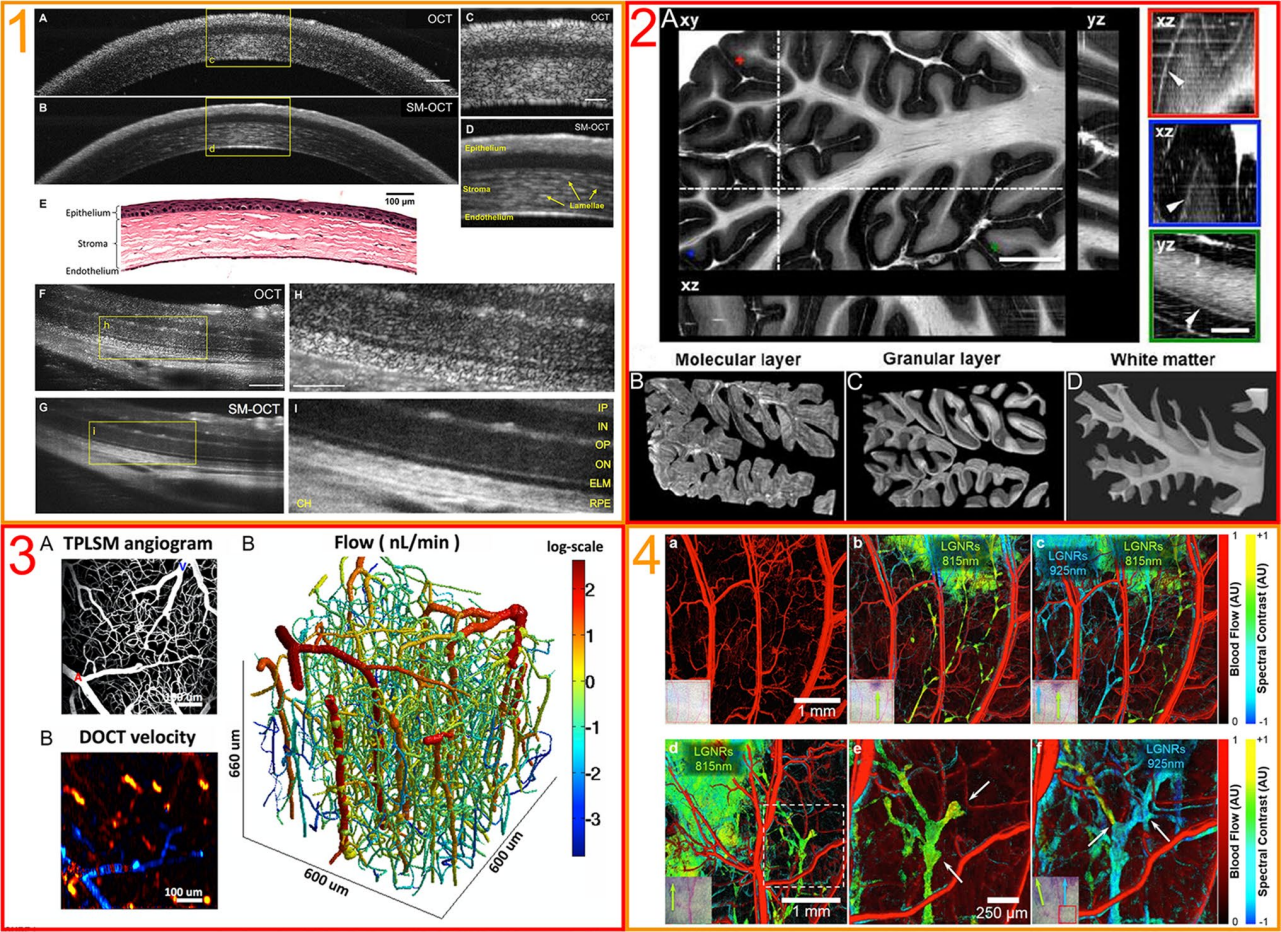
**Recent technical developments in OCT**. (1) Speckle-modulated OCT. Speckle artifacts limit the spatial to noise ratio in OCT imaging. These exemplary speckle-modulated OCT scans of the mouse cornea and retina show the increase of resolution in contrast to conventional OCT imaging. (**1A**) Conventional OCT scan of mouse cornea. (**1B**) Speckle modulated OCT scan of same mouse cornea, notice enhanced sectioning of histological layers. (**1C, D**) Enlarged excerpts (**1D**) notice enhanced delineation of histological structures like lamellae and enhanced delineation of the endothelium in speckle-modulated OCT. (**1E**) Histological section of cornea. (**1F**) Conventional OCT scan of mouse retina. (**1G**) Speckle-modulated OCT scan of mouse retina. (**1H, I**) Enlarged excerpts (**1E**) notice enhanced segregation of single retinal layers; see Yecies et al. [41]. (2) Polarization-sensitive OCT (ps-OCT). Through a set of hardware and software components, polarization-sensitive OCT (ps-OCT) is able to measure and correct the birefringence (“bi-refraction” of light) of local regions of tissue, leading to enhanced imaging of tissue with different optical densities and refraction indices. (**2A**) ps-OCT of a block of human cerebellar lobule. The folded cerebellar cortex is shown on orthogonal viewing planes (**xy** coronal; **xz** axial; **yz** sagittal). Note the ability to delineate the Purkinje cell layer. Volume rendering of segmented (**2B**) molecular layer, (**2C**) granual layer, and (**2D**) white matter (see Wang et al. [37]. (3) Doppler OCT. In vivo delineation of mouse cortical vasculature with Doppler OCT. (**3A**) Multi-photon laser scanning microscopy (MPM) of cerebral vasculature (**3B**) three-dimensional reconstruction of flow demonstrating the vasculature of the mouse cortex. (**3C**) Doppler OCT velocity projection map (see Gagnon et al. [13]. (4) Sensitivity contrast-enhanced OCT**.** Imaging of lymph vessels in ears pinnae in living mice. Injection of large gold nanorods LGNR is used for functional imaging. (**4a**) Delineation of blood vessels (red) by flow detection in OCT prior to LGNR injection. (**4b**) Injection of 815 nm LGNRs (green) and 925 nm LGNRS (cyan). (**4c**) Drainage of LGNRs and delineation of lymphatic vessels. (**4d**) Same imaging technique in a different mouse after injection of LGNRs (**4e**) enlarged excerpt displaying the relationship of blood and lymphatic vessels (**4f)** same area as in (**4e**) after injection of 925 nm LGNRs displaying the (arrow) junction of lymph vessels and mono directional flow (see Liba (2016))

In the neuroimaging domain, recent optical and image processing advancements like automatic serial sectioning of polarization-sensitive OCT (asPSOCT) and speckle modulation even increased image quality to such an extent that in vitro representation of cortical layers at single cell width were possible (see Fig. 1) [13, 29, 37, 41].

OCT has the ability to perform an “optic biopsy”. Not only white and gray matter but also healthy and diffusely invaded brain tissue could be distinguished in glioma surgery [2, 12, 14, 24, 31].

A part from structural imaging, functional brain imaging is possible. Adaptations of perfusion-dependent OCT offer the possibility of functional cortical mapping after peripheral stimulation and furthermore the delineation of epileptic foci [32, 33, 35].

These versatile strengths shed light on OCTs potential for microneurosurgical guidance. This critical literature review focuses on clinical “near” future applications to further enhance neurosurgical excellence.

## Materials and methods

“Pub Med”, “Cochrane Library”, “Crossref Metadata Search”, and “IEEE Xplore” databases as well as the search engine “Google Scholar” were screened for “optical coherence tomography + neurosurgery”, “optical coherence tomography + intraoperative imaging + neurosurgery”, and “microscope integrated optical coherence tomography + neurosurgery”.

## Results

Detailed evaluation of the results revealed *n* = 51 articles related to the use of OCT as an imaging technique in neurosurgery or in the field of neurosurgical research. *n* = 7 articles documented the intraoperative use of OCT in patients. *n* = 4 articles documented the use of microscope-integrated optical coherence tomography as a neurosurgical guidance tool.

## Discussion

### Fundamental research

OCT allows to study microanatomy in vivo. Analysis of the human subarachnoid space with microscope-integrated OCT could delineate for the first time its intact microstructural composition. The arachnoid barrier cell membrane, trabecular system, inlying blood vessels, pia mater, and brain cortex could be well-delineated. OCT was further the first imaging modality to measure the height of these structures in vivo with an accuracy of 7.5 µm. Increased heights of the arachnoid barrier cell membrane at the Sylvian fissure manifested [17] (see Fig. 1).

Analysis of the cranial dura mater demonstrated differentiation of the outer periostal and inner meningeal layer as well as the microanatomical structure of mayor dural blood vessels like arteria meningea media with its vessel wall layers. Measurements of the cranial dura mater documented interindividual highly variable thicknesses [15] (see Fig. 1).

Extravascular OCT could delineate the microstructural composition of cerebral vessel walls with richness of detail [8, 38, 42]. It proved to delineate tunica interna, media, externa, and adventitia in cerebral arteries. Clinical relevant pathologies like calcifications and arteriosclerosis could be further displayed. Scanning of an incidental vasospasm could well define contraction of tunica media with increased thickness and decreased luminal diameters (preliminary results of our research group). The amorphous character of cerebral aneurysm walls with residual tunica media could be delineated for the first time [18] (see Fig. 1).

### Intra-axial lesions

Primary applications of OCT in neurosurgery focused on the ability to distinguish healthy and tumor-infiltrated brain tissue. This ability could be demonstrated in glial tumors as well as malignant melanomas in vitro and later in vivo [2, 4, 5, 24, 27, 34]. Further technical development of cross-polarization OCT even enhanced such tumor detection qualities [22, 39, 40]. Aside from experimental setups, OCT proved these abilities during human glioma resection [1, 14]. Factors which might limit the use of OCT during high-grade glioma surgery might be extended globe shaped resection cavities which need multiple adaptations of the microscope angle to acquire reliable orthograde scans. Another factor could be the diffuse infiltrative growth which often leads to functional-based rather than tumor margin-based resections. Thirdly, 5-ALA states a well-established tool in this domain {Stummer:2006ib}.

### Needle interventions

Due to its physical properties, integration into optical devices is fairly simple [5]. Needle integration aided and controlled the placement of epidural catheters in a porcine model [23]. A lateral viewing probe could discriminate blood vessels at biopsy site in human brain tumors [34]. Since OCT can distinguish between gray and white matter, fine placement of electrodes for deep brain stimulation could be guided in a rodent model [28, 30]. A combination with laser ablation systems has the ability of direct real-time feedback to guide the ablation process in a porcine brain tumor model [7, 11, 21]. These yet experimental theranostic instruments could be promising in the advancing field of minimal invasive tumor, radiant necrosis, and epileptic surgery.

### Extra-axial lesions

OCT-guided dissection of cranial dura mater showed the ability to discriminate dural layers. Thin dura mater in combination with low optical density enabled transdural OCT scanning. These scans showed a sufficient image quality to delineate concealed microanatomical structures like the subarachnoid space, inlying blood vessels, or the brain cortex [15] (see Fig. 1). No literature exists on the use of OCT during meningioma surgery. The above mentioned study suggests that OCT would only be suitable to delineate crucial venous structures like the sinus in certain meningiomas with a low optical density.

### Vascular neurosurgery

**Fig. 2:**
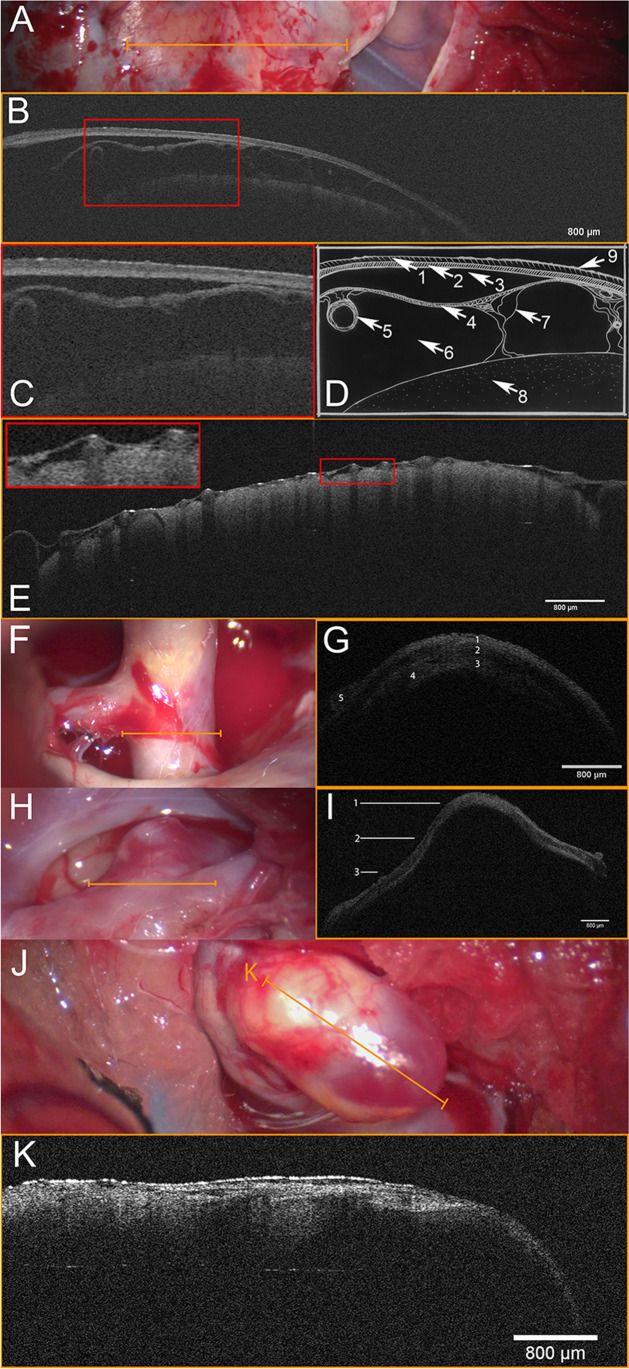
Microscope Integrated OCT. **A** Light microscopic image after right fronto-lateral craniotomy, during dissection of dura mater. Opened segment shows Sylvian fissure with superficial Sylvian veins and temporal as well as frontal brain cortex. Orange line indicates region of scan. **B** OCT scan of dura mater depicting the (**1**) outer endosteal and (**2**) inner meningeal layer. Strikingly, a (**3**) subdural space is present, enabling a clear definition of (**2**) the inner meningeal dural layer and the (**4**) arachnoid barrier cell membrane. Furthermore, (**5**) subarachnoid blood vessels, (**6**) subarachnoid space, (**7**) trabecular system, (**8**) brain cortex, and (**9**) reflection artifacts are depicted by the transdural OCT scan. Red line indicates the area of enlarged excerpt. **C** Enlarged excerpt demonstrating details of transdural OCT scan. **D** Schematic drawing of microstructures: (**1**) + (**2**) dura mater, (**1**) outer endosteal layer, (**2**) inner meningeal layer, (**3**) subdural space, (**4**) subarachnoid space (**4**) arachnoid barrier cell membrane, (**5**) subarachnoid blood vessels, (**6**) subarachnoid space, (**7**) trabecular system, (**8**) brain cortex, and (**9**) reflection artifacts; see Hartmann et al. [16], figure edited with permission from the authors. **E** OCT scan of frontal lobe at frontal operculum visualizing the collapsed SAS after CSF release, with adjacent internal blood vessels. Red rectangle shows enlarged details of the OCT-Scan; see Hartmann et al. [17, 18], figure edited with permission from the authors. **F** Light microscopic intraoperative image of parent vessel: right internal carotid artery. Orange horizontal line indicates area of OCT scan. **G** OCT scan of parent vessel. (**1** Tunica externa; (**2**) tunica media; (**3**) tunica interna; (**4**) atherosclerotic plaque; (**5**) vasa vasorum. **H** Light microscopic intraoperative image of ramus communicans aneurysm seen from a left fronto-lateral approach. **I** OCT scan of the neck of the ramus communicans anterior aneurysm (CA) demonstrating the continuous fading transition from a 3-layered configuration of the parent vessel to the mono-layered appearance of the CA dome. (**1**) CA dome; (**2**) CA neck; (**3**) parent vessel. **J** Light microscopic intraoperative image of right proximal internal carotid artery aneurysm seen from a right fronto-lateral approach; orange lines indicate the area of OCT scan at the aneurysm dome with artherosclerotic plaque. **K** Longitudinal OCT scan at aneurysm dome demonstrating intra-aneurysmatic atherosclerotic plaque; see Hartmann et al. [17, 18], figures edited with permission from the authors

Microscope-integrated OCT could well delineate the microstructural composition of cerebral vessel walls [8, 38, 42] (Fig. 2). Clinical relevant characteristics like wall thickness, different layers, calcifications, and arteriosclerosis could be clearly defined in cerebral arteries, veins, and aneurysms [18]. These promising results should lead to further studies in the field of neurovascular surgery like bypass surgery [20].

### Arachnoid cyst

Microscope-integrated OCT could demonstrate the membrane of a middle fossa cerebral arachnoid cyst. Transcystic OCT at site of the temporal lobe delineated the trabecular system of the arachnoid space, inlying cerebral arteries and veins as well as the brain cortex. At site of fenestration, OCT excluded hidden crucial anatomic structures prior to the dissection of the membrane [16].

### Peripheral nerves

Intraoperative handheld OCT during peripheral nerve surgery could delineate single bundles of nerve fascicles [6]. Image quality was influenced by motion artifacts and wrapping of the imaging probe with sterile foil. Microscope-integrated systems would eliminate these restraints and improve the surgical work flow. Clinical relevant data which correlates the rehabilitation potential with intraoperative OCT similar to the work published on optic nerve rehabilitation are missing {Wilson:2020br}.

## Conclusion

Intraoperative OCT offers the possibility to study **microanatomy in vivo** approaching the resolution of conventional histology. Manifold applications could deepen our physiological and pathophysiological understanding; e.g., in case of the choroid plexus, OCT videos could elicit mechanisms of liquor production in correlation to blood pulse.

Data which proves that microscope-integrated OCT lowers the perioperative morbidity or extent of tumor resection does not exist.

Experience from our group suggests that the regular use of microscope-integrated OCT could increase security during certain critical surgical steps. In case of dural dissection during transtentorial approaches, tumor resection at mayor venous blood vessels like sigmoid sinus, removal of craniopharyngiomas, transsulcal preparation, and dissection of the Sylvian fissure - OCT could delineate crucial structures prior to dissection. Here, augmented reality is needed for intuitive integration into the microsurgical workflow.

For microsurgical considerations, it is worth noting that valuable OCT scanning is only possible if the surgical trajectory exposes the pathology in an orthograde scanning angel. This general principle of microscope-integrated OCT is of particular significance during key hole surgery or other narrow approaches, e.g., to ramus communicans anterior aneurysms or supracerebellar infratentorial approaches to the pineal region.

Integration of OCT in **endoscopy** could aid surgeries with no access for real-time imaging methods like sonography. In case of hypophyseal surgery, OCT might define concealed hypophyseal arteries, cavernous sinus walls, and inlying structures as well as tumor and hypophyseal tissue to extend resection while lowering perioperative morbidity.

Combinations of OCT and minimal invasive **needle devices** seem to hold promise in tumor surgery. Biopsy needles with integrated forward and lateral viewing probes could lower perioperative morbidity by securing blood vessels and functional relevant brain structures as well as control biopsy positioning. Combination of OCT and laser ablation further offers the possibility to perform “optic biopsies” and adapt the coagulation process in real time. In the emerging field of minimal invasive surgery, these systems might gain further relevance.

OCT offers unprecedented quality to delineate the microstructural composition of **vessel walls and their pathologies**. In aneurysm surgery, OCT of the neck of the aneurysm could help to aid clip placement in relation to intravascular arteriosclerosis, thrombosis, aneurysm wall thickness, and vessel wall calcifications—characteristics and pathologies which were concealed so far. In case of **bypass surgery** OCT would be an imaging method which could aid to determine optimal site of bypass in correlation to vessel wall pathologies.

Neuroscientific advancements like diffusion-dependent OCT for **functional brain imaging** have not yet found clinical applications. Brain pulsation and vessel artifacts as well as intermodality validation with hemodynamic and electrophysiological measurements still inhibit clinical transfer [32].

Spatial resolution of polarization-sensitive OCT lays yet **beyond the scope of manual microsurgery**. If robotic surgery further develops, OCT might gain novel importance as a real-time distance measuring tool.

## Data Availability

Not applicable for a review. All citations in the text and figures are given.

## Code Availability

Not applicable for a review.
